# Neuroendocrine Mechanisms of Acupuncture

**DOI:** 10.1155/2012/792793

**Published:** 2012-07-10

**Authors:** Fengxia Liang, Rui Chen, Edwin L. Cooper

**Affiliations:** ^1^Acupuncture and Moxibustion Institute, College of Acupuncture and Moxibustion, Hubei University of Chinese Medicine, Wuhan 430061, China; ^2^Department of Integrated Traditional Chinese Medicine and Western Medicine, Union Hospital, Tongji Medical College, Huazhong University of Science and Technology, Wuhan 430022, China; ^3^Laboratory of Comparative Neuroimmunology, Department of Neurobiology, David Geffen School of Medicine at UCLA, University of California, Los Angeles, CA 90095-1763, USA

Acupuncture is currently gaining popularity worldwide as a “complementary” or “alternative therapy.” The underlying mechanisms of acupuncture in general require further investigation to be delineated, although acupuncture therapy has been demonstrated to be effective in several clinical areas. Recently, there is a growing focus on the critical role of the brain and a need to explain how acupuncture affects endocrine function through the CNS (central nervous system). This special issue was developed to stimulate the continuing efforts in defining and promoting the neuroendocrine mechanism of acupuncture.

This special issue contains thirteen papers, of which five are related to analgesic effect of acupuncture, and two cover opiate addiction. There are single papers focusing on cardiac, Parkinson's disease, hot flashes, and hypertension. Another deals with brain-modulated effect of auricular acupressure. Finally, one explores the impairments of spatial memory.

*“Effects of electroacupuncture on N-methyl-D-aspartate receptor-related signaling pathway in the spinal cord of normal rats”* by H.-N. Kim provides evidence that calcium influx by N-methyl-D-aspartate receptor activation may play an important role in EA analgesia of normal rats through modulation of the phosphorylation of spinal phosphatidylinositol 3-kinase (PI3K) and cAMP response element-binding protein (CREB). “*Changes in cytokine expression after electroacupuncture in neuropathic rats”* by M. H. Cha revealed that EA reduced the levels of proinflammtory cytokines elevated after nerve injury in peripheral nerves and dorsal root ganglia (DRG). “*Effects of electroacupuncture at BL60 on formalin-induced pain in rats”* by K.-H. Chang showed the effect of EA in relieving inflammatory pain and the possible involved mechanism. Furthermore, “*Effect of electroacupuncture on activation of p38MAPK in spinal dorsal horn in rats with complete Freund's adjuvant-induced inflammatory pain” *by Y. Liang indicated that anti-inflammatory and analgesic effect of EA might be associated with its inhibition of spinal p38 MAPK activation and thereby provide a potential mechanism for the treatment of inflammatory pain by EA. On the other hand, “*Does acupuncture needling induce analgesic effects comparable to diffuse noxious inhibitory controls*?” by J. Schliessbach showed that acupuncture at low pain stimulus intensity did not produce a DNIC-like effect comparable to a classical, painful DNIC-test, indicating that the penetration of an acupuncture needle seems not to induce an analgesic effect mainly mediated by DNIC.

*“Acupuncture for the treatment of opiate addiction”* by J. G. Lin is a systematic review of randomized clinical trials which applied acupuncture for treating opiate addiction and analysed the possible mechanism underlying the effect of acupuncture. “*Electroacupuncture suppresses discrete cue-evoked heroin-seeking and Fos protein expression in the nucleus accumbens core in rats” *by S. Liu highlights the therapeutic benefit of EA in preventing relapse to drug addiction, through the results that EA stimulation reduced active responses elicited by discrete cues and attenuated Fos expression in the core but not the shell of the nucleus accumbens. “*Electroacupuncture at PC6 (Neiguan) improves extracellular signal-regulated kinase signaling pathways through the regulation of neuroendocrine cytokines in myocardial hypertrophic rats”* by J. Li revealed that EA could improve cardiac function in rats with myocardial hypertrophy by modulating upstream neuroendocrine cytokines that regulate extracellular signal-regulated kinase (ERK) signaling. This proposes a mechanism underlying EA's effect in treating cardiac diseases.

The cortical and striatal gene expression profile of 100 Hz electro-acupuncture treatment in 6-hydroxydopamine-induced Parkinson's disease model by Li-Rong Huo applied high-throughput microarray analysis to analyze gene expressions. This study suggested that EA may induce recovery of homeostasis in the transcript network and many regulated functional clusters in the cortex and striatum; this characteristic underlies the mechanism of EA's effect in improving behavioral characteristics of PD rats. “*Acupuncture as treatment of hot flashes and the possible role of calcitonin gene-related peptide”* by A.-C. S. Holm discussed the role of CGRP involved in acupuncture as an alternative treatment for hot flashes, based on the evidence for connections between the opioid system and the release of CGRP. *“Neuroendocrine mechanisms of acupuncture in the treatment of hypertension”* by W. Zhou discussed current knowledge of acupuncture effects on central nervous system and how they contribute to regulation of acupuncture on the endocrine system. This approach provides a perspective on treating of hypertension. *“Brain-modulated effects of auricular acupressure on the regulation of autonomic function in healthy volunteers”* by X. Gao investigated the acute effect of ear acupressure on autonomic function, indicating that this approach of auricular acupressure was based on intensification of the related mechanism of blood pressure regulation. *“Acupuncture stimulation alleviates corticosterone-induced impairments of spatial memory and cholinergic neurons in rats”* by B. Lee demonstrated that stimulation of HT7 acupoint produced significant neuroprotective activity against the neuronal impairment and memory dysfunction by immune responses and gene expression.

Due to recent development not only in invasive methodology such as PET in human and animals and optogenetic technique, but also in molecular biology, the research of acupuncture at the whole organismic level and an in-depth analysis becomes more available. It is essential to focus on some critical factors which impact the effect of acupuncture, such as biophysical action of acupoints, combination of acupoints, and acupuncture method. A recent study proposed a neurophysiological mechanism to explain the beneficial effects of acupuncture based on the stimulated purinergic signalling by acupuncture. This potential provokes some scientists interested in acupuncture to investigate further in this rapidly expanding field. Finally, we should never forget the need for careful consideration of the role of placebo in this and other CAM analyses.

Of course, the selected topics and papers are not a comprehensive representation of the area of this special issue. Nonetheless, they represent the rich and many-faceted knowledge that we have the pleasure of sharing with the readers.

